# Inflammatory Fibroid Polyp: An Unusual Cause of Abdominal Pain in the Upper Gastrointestinal Tract a Case Report

**DOI:** 10.1515/med-2020-0033

**Published:** 2020-03-25

**Authors:** Huan Wang, Tiejun Zhou, Cuiwei Zhang, Hao Li, Muhan Lü

**Affiliations:** 1 Department of Gastroenterology, The Affiliated Hospital of Southwest Medical University, Luzhou, Sichuan, China; 2 25 Taiping Road, Luzhou,Sichuan Province 646000, People’s Repubic of China; 3 Department of Pathology, The Affiliated Hospital of Southwest Medical University, Luzhou, Sichuan, China

**Keywords:** Vanek’s tumor, Endoscopy, Pathology, Immunocytochemistry

## Abstract

Inflammatory fibroid polyps (IFPs) tend to occur in the gastrointestinal tract, and they are rare and benign neoplasms. In general, IFPs often come from epithelial tissue. The gastric antrum is the most common location. Endoscopic ultrasound (EUS) often shows a predominantly hypoechoic mass with well-defined borders originating from the submucosal area. Here, we report the case of a 46-year-old woman with abdominal pain who underwent computed tomography (CT), endoscopic ultrasound and endoscopic submucosal dissection (ESD) of resected specimens; the diagnosis was ultimately an inflammatory fibroid polyp. She is currently in clinical remission.

## 1 Introduction

The IFPs are rare benign mesenchymal tumors that can arise in the gastrointestinal tract. In general, IFPs often come from epithelial tissue, and always located in the gastric antrum [1]. IFPs have been reported to cause abdominal pain, anemia, gastrointestinal tract bleeding, weight loss, and vomiting [2]. EUS often shows a predominantly hypoechoic mass with well-defined borders originating from the submucosal area. It also contains a proliferation of small blood vessels. The inflammatory infiltrate contained lymphocytes and plasma cells. IFPs are often confused with some submucosal lesions, such as gastric stromal tumors (GISTs) or a heterotopic pancreas. In this case, we reported on a middle-aged woman with IFP. It is extremely rare in the world [3].

## 2 Case report

**Figure 1:**
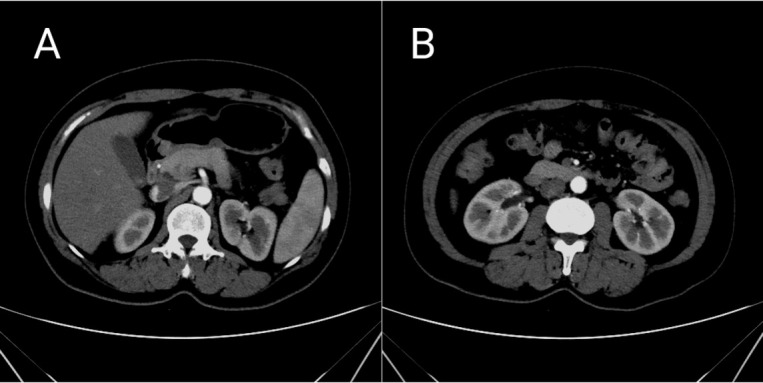
CT Abdomen with IV Contrast The coronal image of contrast-enhanced CT scan revealed that the lesion was within normal limits, and the gastric wall was poor filling. On transverse, the intestinal tract is normal. There was no sign of ileus. CT= Computed Tomography; IV= intravenous

**Figure 2:**
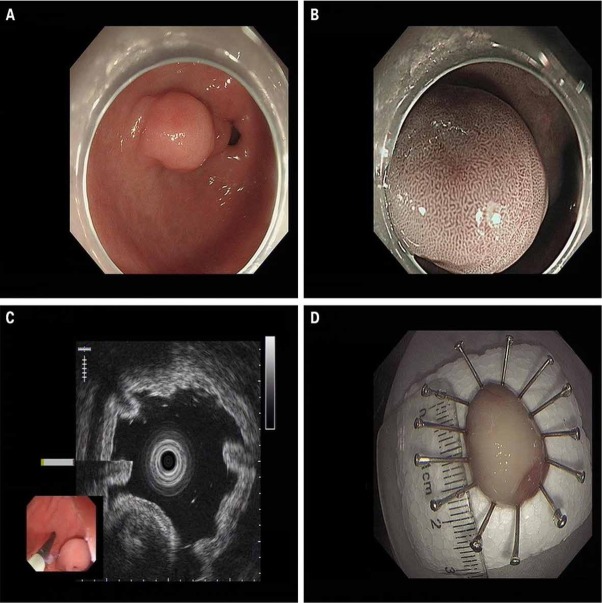
Endoscopic performance A. A submucosal protuberant lesion in the gastric antrum with well-defined borders. B. Surface microstructure and microvascular patterns were regular. Its surface appearance was similar to that of the surrounding normal tissue. There seemed to be a slight depression in the center of the lesion. The glands were slightly dense. C. A hypoechoic tumor with slightly heterogeneous internal echoes originating from the submucosal area located at the gastric antrum. D. Pathological tissue obtained by ESD, which was a yellow oval tissue measuring 1.3*1.0 cm. ESD= Endoscopic submucosal dissection

**Figure 3:**
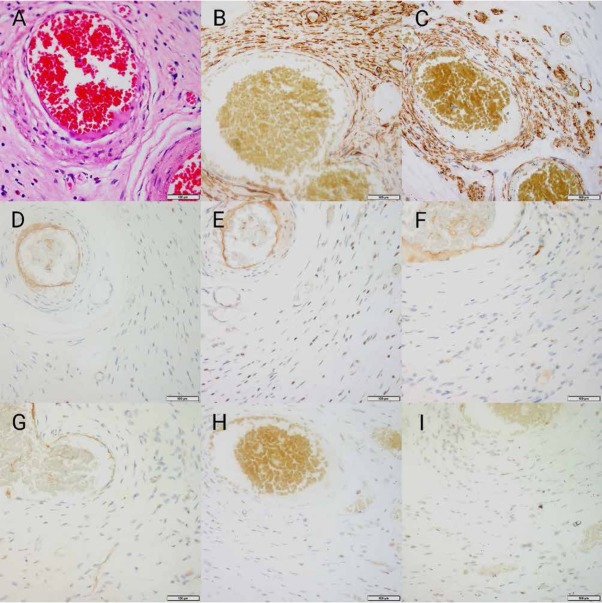
Histopathologic and immunohistochemical expression of pathological tissue. HE×400. The tumor cells demonstrated fusiform, vortex-like and onion-like hyperplasia around the blood vessels, there were some eosinophils and lymphocytes, and a small number of plasma cells infiltrated the stroma. CD34. Immunohistochemical findings revealing spindle cells stained positive for CD34(tumor cells +) × 400. SMA. Immunohistochemical findings revealing spindle cells stained positive for SMA (tumor cells +) × 400. CD117. Immunohistochemical stain for CD117 shows negative in the lesional cells(tumor cells +) × 400. DOG-1. Immunohistochemical stain for DOG-1 shows negative in the lesional cells(tumor cells +) × 400. S100. Immunohistochemical stain for S100 shows negative in the lesional cells(tumor cells +) × 400. ALK. Immunohistochemical stain for ALK shows negative in the lesional cells(tumor cells +) × 400. P53. The P53 labeling index was 1%(tumor cells +) × 400. Ki-67. The Ki-67 labeling index was 5% in the spindle cells(tumor cells +) × 400. HE= Hematoxylin-eosin staining, SMA= SmoothMuscle Actin

A 46-year-old woman presented with continuous abdominal pain for more than a month. There was no family history of gastrointestinal tumors. There was no sign of peritonitis, and no abnormal findings were detected from the laboratory studies performed at admission, such as serum electrocytes, TB-IFN, and the immune PHOMO check. The CT results were within normal limits (Fig.1). The EUS indicated a hypoechoic tumor with slightly heterogeneous internal echoes originating from the submucosal area located at the stomach antrum, measuring 1.3*1.0 cm with well-defined borders (Fig.2A BC). The tumor protruded into the cavity. Upon ESD, we could see a yellow oval tissue with a well-defined border that was similar to fat or pancreatic lesions (Fig.2D). Histopathologic analysis indicated an onion-skin-like concentric formation of the fibroblastic stroma and spindle cells with inflammatory cell infiltration, predominantly by numerous eosinophils (Fig.3A). Immunohistochemical examination showed that the cells were positive for CD34 (Fig.3B), Smooth Muscle Actin (SMA) (Fig.3C), and were negative for CD117 (Fig.3D), DOG-1 (Fig.3E), S100 (Fig.3F) and ALK (Fig.3G). The P53 labeling index was 1% (Fig.3H), whereas the Ki-67 labeling index was 5% in the spindle cells (Fig.3I). The histopathological and immunohistochemical findings were consistent with an IFP. By using an acid inhibitor and painkiller, the abdominal pain was not significantly improved, but was relieved after ESD. She is currently in clinical remission. Finally, we diagnosed the tumor as an IFP because the histopathological and immunohistochemical findings were the same.

**Ethical approval**: The research related to human use has been complied with all the relevant national regulations, institutional policies and in accordance the tenets of the Heisinki Declaration and has been approved by the Affiliated Hospital of Southwest Medical University review board.

**Informed consent**: Informed consent has been obtained from patient included in this study.

## 3 Discussion

**Figure 4:**
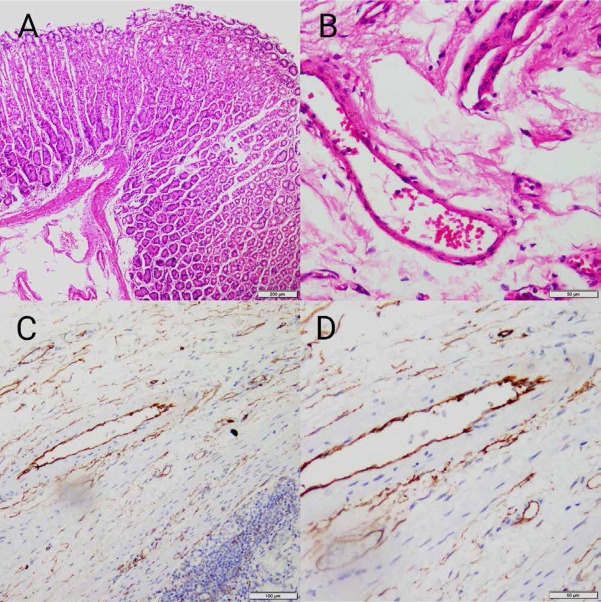
Histopathologic and immunohistochemical expression of normal tissues. A. HE×40. Covered with maturely differentiated mucosal epithelium. The submucosa consisted of loose fibrous connective tissue. B. HE×400. The wall of small vessels in the submucosa was thin and surrounded by fibrous cells and adipocytes. C. CD34. Immunohistochemical findings revealing vascular endothelial cells stained positive for CD34(normal cells +) × 100. D. CD34. Immunohistochemical findings revealing vascular endothelial cells stained positive for CD34(normal cells +) × 400. HE= Hematoxylin-eosin staining

The first description of this rare finding of a “polypoid fibroma” by Konjetzny occurred in 1920. The term “IFP” was first purposed by Helwig and Rainer in 1953 [3]. The IFP is now generally accepted and regarded as different from eosinophilic gastroenteritis and other conditions with which it had been previously confused. Lesions can present at any age and have been recorded as isolated except in one case [4]. Multiple and recurrent inflammatory fibroid polyps in three generations of a Devon family were reported in 1984 [5]. The aetiology remains unknown. IFPs are benign lesions with a low incidence which are not easily transferred and rarely recur. The clinical symptoms are numerous and complex depending on the location of the polyp and the age of the patient. Thus, auxiliary inspections are often needed. X-rays, US, CT and EUS are used to reach a diagnosis [6]. The cause of abdominal pain cannot be ruled out as ileus, but the CT results were within normal limits, so ileus was not considered. According to the results that the EUS showed, we wondered whether the tumor was a heterotopic pancreas, a GIST or an IFP [7]. With EUS, a heterotopic pancreas has uneven echogenicity and often originates from the submucosal area or the muscularis propria; it is always located in the antrum. Gastric polyps usually originate from the mucosal layer, and IFPs originate from the submucosal area. A GIST often predominantly demonstrates a hypoechoic mass with EUS, but the appearance can change due to calcification or liquefaction necrosis. At the same time, it is often located in the submucosa and originated from the muscularis propria. It has a certain invasiveness and can be transferred, while there are still some IFP cases reported to be invasive though in a rareness [8]. The performance of a GIST under endoscopy is too similar to that of our case [9]. Therefore, we performed histopathological and immunohistochemical examinations. However, differentiation is difficult, especially between IFPs and GISTs. In IFPs, vessels are usually circled by a characteristic concentric arrangement of fibroblasts with an onion-like appearance [10]. GISTs are intramural tumors that usually lack perivascular concentric cuffing. Immunohistochemical examination also plays an important role in distinguishing normal tissue from tumor tissue and can confirm the limited diagnosis (Fig.4). Both of these tumors are usually positive for CD34, but only GISTs are positive for DOG-1 and CD117 [11]. The final diagnosis was an IFP [1].

Endoscopic polypectomy rarely results in recurrence, and the prognosis is favorable when the polypoid lesion is no more than 2 cm; however, it may result in perforation

or incomplete resection, increasing the chance of local recurrence [12]. Currently, surgery is the main treatment [13]. Usually, a complete resection of IFPs is needed [2]. EUS can determine the depth of the lesion to help assess the choice of treatment. Hence, we performed ESD for the patient who is currently in clinical remission.

IFP is a rare disease in the digestive tract. Some studies have been commissioned to explore the mechanism of IFP all the time. Immunohistochemically, the lesional mesenchymal cells express CD34 and platelet-derived growth factor receptor alpha (PDGFRA). Most IFPs bear PDGFRA mutations [14, 15]. In recent years, some studies show that IFP is a benign tumor with PDGFRA gene activation mutation [16]. The understanding of the role of PDGFRA in the pathogenesis of IFP will provide a theoretical basis for further study of tumors with the PDGFRA gene mutation and clinical targeted therapy.

## 4 Conclusion

The IFP was identified and diagnosed by histopathological, immunohistochemical examination and EUS.
